# Hepatitis B and C virus prevalence in a rural area of South Korea: the role of acupuncture

**DOI:** 10.1038/sj.bjc.6600436

**Published:** 2002-08-01

**Authors:** H R Shin, J Y Kim, J I Kim, D H Lee, K Y Yoo, D S Lee, S Franceschi

**Affiliations:** Division of Cancer Control and Epidemiology, National Cancer Center Research Institute, 809 Madu-dong, Ilsan-Gu, Koyang, Kyonggi, 411-764 South Korea; Department of Preventive Medicine, College of Medicine, Dong-A University, 1-3 Dong-Dae-Shin-Dong, Seo-Gu, Busan, 602-714, South Korea; Department of Preventive Medicine, College of Medicine, Kosin University, 34 Am-Nam-Dong, Seo-Gu, Busan, 602-702, Korea; Department of Preventive Medicine, College of Medicine, Seoul National University, 28 Yongon-Dong, Chong-No-Gu, Seoul, 110-799, South Korea; Department of Clinical Pathology, College of Medicine, Seoul National University, 28 Yeon-Keon-Dong, Chong-No-Gu, Seoul, 110-799, South Korea; Unit of Field Intervention Studies, International Agency for Research on Cancer, 150 Cours Albert Thomas, F-69372 Lyon cedex 08, Lyon, France

**Keywords:** hepatitis C virus, hepatitis B virus, acupuncture, blood transfusion, South Korea

## Abstract

A cross-sectional study evaluated the prevalence of and the risk factors for hepatitis C and B viruses among 700 adults above the age of 40 years in a rural area of South Korea. Seropositivity for hepatitis C virus antibody (11.0%, 95% confidence interval: 8.7–13.6) was higher than that for hepatitis B surface antigen (4.4%, 95% confidence interval: 3.0–6.2). Anti-hepatitis C virus seropositivity was associated with a history of repeated acupuncture (odds ratio=2.1, 95% confidence interval: 1.1–4.0), and blood transfusion (odds ratio=5.5, 95% confidence interval: 1.6–19.3) before 1992 when hepatitis C virus screening in blood donors became mandatory. Hepatitis C virus 2a was the most prevalent genotype, followed by 1b. Hepatitis C virus risk attributable to acupuncture was 38% (9% for men and 55% for women). Safer acupuncture practice has become a priority for hepatitis C virus prevention in South Korea.

*British Journal of Cancer* (2002) **87**, 314–318. doi:10.1038/sj.bjc.6600436 www.bjcancer.com

© 2002 Cancer Research UK

## 

Liver cancer mortality in South Korea is one of the highest in the world at 30.3 and 9.7 per 100 000 for men and women, respectively (world age-standardised Who, 1998). Both hepatitis B and C viruses (HBV and HCV) infection are strong risk factors for the development of liver cancer in South Korea (Shin *et al*, 1996), as well as in other countries (Iarc, 1994). In 1991, HBV was found to be the most important risk factor for hepatocellular carcinoma (HCC) in Korea (Yoo *et al*, 1991). Since then, however, HCV infection has shown a stronger association in elderly HCC patients (Lee *et al*, 1993; Huh *et al*, 1998).

Since the introduction of an HCV antibody (anti-HCV) test for screening blood donors in Korea in April 1991, HCV exposure among blood recipients has decreased. However, HCV transmission may still be prevalent in certain areas, due to such practices as acupuncture. Indeed, a history of acupuncture has been reported by more than one third of subjects in a study in South Korea (Shin *et al*, 2000). Major indications for acupuncture include arthritis and other joint diseases, the sequelae of trauma, headache, and a broad range of minor diseases not responding to conventional medical treatments (http://www.acu.pe.kr).

A survey in 1993 in a rural district showed a high prevalence of anti-HCV (5.5% among subjects over 10 years of age) and suggested a role for parenteral viral exposure other than blood transfusion (Shin *et al*, 2000). We therefore conducted a cross-sectional study in a different part of the same rural area.

## METHODS

### Study area and subjects

The study area was in a rural part of south-east Korea (population 4500) where mortality rates for liver cancer are 120% and 50% higher than the national average in men and women, respectively (Lee *et al*, 2001). The majority of the population in the study area is employed in agriculture, and their socio-cultural characteristics are similar to those in other rural areas in Korea.

In 1999, the 2299 inhabitants of the area over 40 years of age formed the target population for a survey in the preparation for a large prospective cohort study (Yoo *et al*, 2002). The survey aimed to assess the prevalence of, and risk factors for, infection with HBV, HCV and Clonorchis sinensis. Subjects were not individually invited, but were made aware of the survey by local health authorities and village heads (e.g., through village meetings and local broadcasting). Seven hundred subjects gave their informed consent and underwent the survey (288 men and 412 women, comprising 30.2% and 31.8% of male and female residents, respectively). Among these were 146 couples thereby allowing study of heterosexual transmission of HCV and HBV between spouses.

A structured questionnaire was completed for each individual by trained interviewers covering demographic characteristics, medical history including blood transfusion, acupuncture, smoking, and drinking habits.

The total frequency of blood transfusion and acupuncture sessions was evaluated. As screening for HCV among blood donors started in Korea in April 1991, the study participants were asked to report the year in which they first received blood transfusion and also acupuncture. Alcohol drinking was defined as regularly consuming at least one alcoholic drink per month. Daily intake of more than 80 grams of alcohol for more than 10 years was classified as heavy drinking. Individuals who had smoked more than 20 packs of cigarettes prior to the survey were classified as smokers and those who had smoked more than one pack per day for more than 10 years were classified as heavy smokers.

### Serological study

Serum samples, frozen at −70°C within 2 h of blood collection, were tested for anti-HCV and hepatitis B surface antigen (HBsAg). Anti-HCV was tested using the Abbott enzyme immunoassay (EIA) kit, HBsAg using the EIA method. The liver enzymes, alanine aminotransferase (ALT), and aspartame aminotransferase (AST) were also evaluated.

### Detection and genotyping of serum HCV RNA

Presence of HCV RNA viremia was confirmed by nested reverse transcript-polymerase chain reaction (RT–PCR), using Roche Amplicor, the detection limit being 100 copies per ml. Hepatitis C virus genotypes 1a, 1b, 2a, 2b and 3a were determined by amplification of the core region using genotype-specific primers as described by Okamoto *et al* (1993).

### Statistical analysis

Odds ratios (ORs) and corresponding 95% confidence intervals (CIs) of being seropositive for anti-HCV or HBsAg were computed by means of unconditional multiple logistic regression analysis including age, sex, education, smoking habits, and history of blood transfusion and acupuncture (Sas Institute Inc, 1999). Linear trends in risk according to the increase of exposure were evaluated by likelihood ratio tests for trend based on linear logistic regression models. Adjusted attributable risk fractions were computed (Mezzetti *et al*, 1996).

## RESULTS

### Seroprevalence of anti-HCV and HBsAg

The seroprevalence of anti-HCV (11.0%, 95% CI: 8.7–13.6) was higher than that of HBsAg (4.4%, 95% CI: 3.0–6.2) (Table 1

**Table 1:**
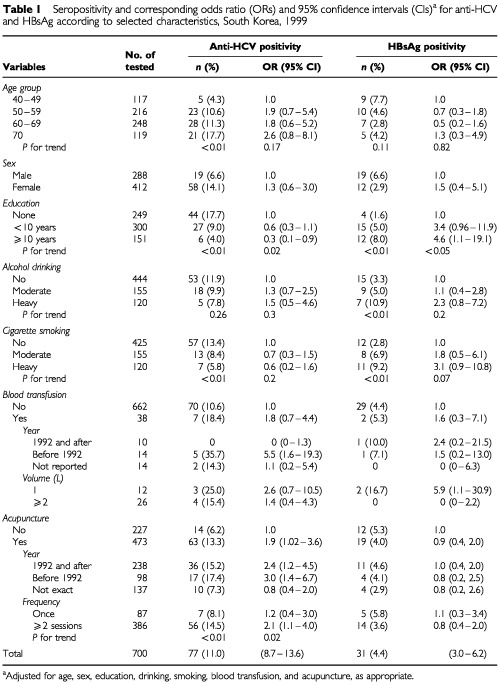
Seropositivity and corresponding odds ratio (ORs) and 95% confidence intervals (CIs)^a^ for anti-HCV and HBsAg according to selected characteristics, South Korea, 1999

). Seropositivity of anti-HCV in women (14.1%) was more than two-folds higher than in men (6.6%). The opposite was found for HBsAg-positivity (6.6% in men, 2.9% in women). Seropositivity of anti-HCV increased with age (*P* for trend <0.001), while seropositivity for HBsAg decreased (*P*=0.001) (Figure 1

**Figure 1:**
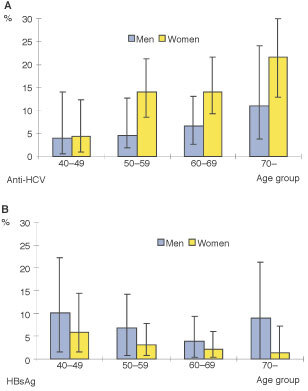
Seropositivity of anti-HCV (**A**) and HBsAg (**B**) among men and women, South Korea, 1999.

).

### Risk factors for HCV infection

The association between seropositivity for anti-HCV and HBsAg and selected characteristics is shown in Table 1. After adjustment for all variables listed in Table 1, the crude OR of anti-HCV in women decreased from 2.3 (95% CI: 1.3–4.0) to 1.3 (95% CI: 0.6–3.0). In contrast, the crude OR of HBsAg in women increased from 0.4 (95% CI: 0.2–0.9) to 1.5 (95% CI: 0.4–5.1).

Whereas education was inversely associated with the risk for anti-HCV seropositivity (OR for more than 10 years of education *vs* none=0.3, 95% CI: 0.1–0.9), it was directly associated with HBsAg seropositivity (OR=4.6, 95% CI: 1.1–19.1). Drinking alcohol was not significantly associated with seropositivity for either anti-HCV or HBsAg. An increased risk of borderline statistical significance was found for HBsAg among cigarette smokers (OR for heavy smoker *vs* none=3.1, CI: 0.9–10.8). A history of blood transfusion was not significantly associated with seropositivity for either anti-HCV or HBsAg.

Compared to individuals who had never been transfused, those with a history of transfusion before 1992 had an increased risk (OR=5.5, 95% CI: 1.6–19.3) for anti-HCV positivity but not for HBsAg positivity (OR=1.5, 95% CI: 0.2–13.0) (Table 1). None of the 10 recipients who were transfused in or after 1992 was positive for anti-HCV, but one subject was seropositive for HBsAg. Only one individual reported having a transfusion more than once. Blood volume was associated with seropositivity for neither anti-HCV nor HBsAg. Whilst 13.3% of subjects with a history of acupuncture were seropositive for anti-HCV, only 4.0% showed HBsAg seropositivity. An association was found between acupuncture and anti-HCV seropositivity (OR=1.9, 95% CI: 1.0–3.6), but not HBsAg (OR=0.9). The increased risk from acupuncture did not vary by year at first exposure. OR for anti-HCV was higher among those who underwent acupuncture twice or more (OR=2.1, 95% CI: 1.1–4.0) than among those who had this only once (OR=1.2) (Table 1).

### HCV RNA genotype

**Figure 2:**
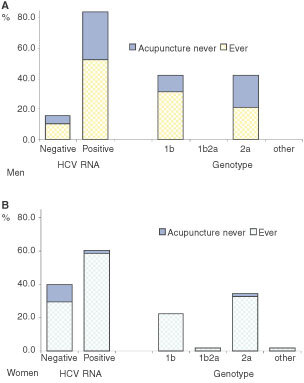
Distribution of HCV seropositive subjects by presence of HCV RNA, genotypes, and acupuncture history in men (**A**) and women (**B**), South Korea, 1999.

HCV RNA was detected by RT–PCR in 51 (66.2%) of 77 anti-HCV positive subjects. The detection rate of HCV RNA among anti-HCV positive subjects was significantly lower in women (58.6%) than in men (84.2%). The most prevalent HCV genotype was 2a followed by 1b. Most subjects with HCV RNA detected had a history of acupuncture (Figure 2

).

### Inter-spousal transmission of HCV infection

In three of 146 couples who took part in this aspect of the study, seropositivity for anti-HCV was found in both partners (Table 2

**Table 2:**
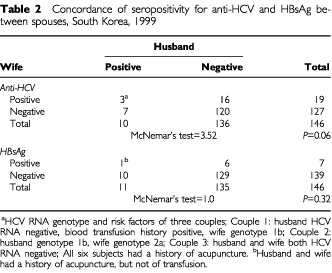
Concordance of seropositivity for anti-HCV and HBsAg between spouses, South Korea, 1999

). In the only couple both partners were HCV RNA positive, HCV genotypes were different. All six members of anti-HCV positive concordant couples had a history of acupuncture. In only one couple were both spouses seropositive for HBsAg, both with a history of acupuncture, but not blood transfusion.

## DISCUSSION

The major findings from our present study in South Korea are (1) a high prevalence of anti-HCV seropositivity, more than double that of HBsAg seropositivity and (2) that acupuncture is an important cause of this. Acupuncture was associated with a doubling in the prevalence of anti-HCV, but not of HBsAg.

As 34.1% of men and 62.9% of women reported having had an acupuncture session more than once, the attributable risk fraction for HCV infection was 38% (95% CI: 8.0–69.5) overall (9.4%, 95% CI: 0.5–67.3 among men and 54.5%, 95% CI: 21.2–87.8 among for women). In contrast, the risk fraction attributable to blood transfusion was 4% (95% CI: −3.2–11.1).

Seroprevalence of HCV infection varies widely between and within countries, ranging from 1–2% in most western countries (Dubois *et al*, 1997; Alter *et al*, 1999; Goritsas *et al*, 2000), and in non-endemic areas in Japan (Kiyosawa *et al*, 1994; Kayaba *et al*, 1998) to more than 15% in Egypt (Frank *et al*, 2000), Italy (Raffaele *et al*, 2001), and in endemic areas of Japan (Kiyosawa *et al*, 1994).

The only efficient route for HCV transmission is parenteral. As the prevalence of blood transfusion in the general population is less than 1%, high-risk areas for HCV are characterised by a relatively high frequency of other parenteral treatments (e.g., intra-muscular injections for minor conditions in Italy before disposable needles and syringes became available, Raffaele *et al*, 2001).

Acupuncture, tattooing, and folk medicines have been associated with an increased risk of HCV seropositivity in several studies from Asia (see Kao and Chen, 2000, for a review), including Japan (Michitaka *et al*, 1991; Kiyosawa *et al*, 1994), Taiwan (Sun *et al*, 1999), Indonesia (Sulaiman *et al*, 1995) and South Korea (Shin *et al*, 2000). The relative risk in people after acupuncture ranged between 1.9 (Michitaka *et al*, 1991) and 3.3 (Shin *et al*, 2000) and tended to increase with the increase in acupuncture sessions. No increased risk of HCV infection after acupuncture, however, was found in Japan, among atomic bomb survivors (Fujiwara *et al*, 2000), the United Kingdom (Neal *et al*, 1994) and the United States (Alter, 1999), though in Western countries the practice was seldom reported (Alter, 1999). The proportion of people with a history of acupuncture in our survey in South Korea (68%) is the highest reported so far in any study.

Our estimate of HCV seropositivity agrees with a previous survey (11% among adults over 40 years old) (Shin *et al*, 2000), although it is higher than in other studies (Kim *et al*, 1992; Kim *et al*, 1997; Seo and Lee, 1998) from South Korea.

Conversely, our present estimate of HBsAg seropositivity is similar to that found by the 1998 National Health and Nutrition Survey in South Korea (Kihsa, 1999). In the mid-1980s a nationwide campaign of HBV vaccination was initiated in the country, the vaccine being mandatory for newborn children and recommended to adults (Ahn, 1996).

Apart from acupuncture and blood transfusion before routine HCV screening of blood donors, only a high educational level was significantly associated with HCV positivity in this study. Interestingly, whereas the excess of HCV positivity in women compared to men disappeared after allowance for acupuncture history, the inverse association with years of education was not modified. In fact, whereas women in South Korea showed a three-fold increased risk to have had acupuncture compared to men, no clear difference in such practice emerged according to educational level.

The association with HCV seropositivity in our survey was stronger among those who underwent acupuncture twice or more than those who had it only once and was, therefore, unlikely to be due to chance. The association with HCV following first acupuncture after 1991 suggested that the transmission risk has persisted in recent years.

Possible bias and confounding factors may be relevant. Selection bias is of concern, as only one third of the population joined our health survey. The prevalence of HCV and, possibly, the association with acupuncture history might have been overestimated in our survey if people with liver disease or those being afraid of the possible dangers of acupuncture had selectively accepted to participate. We had, however, information on liver enzymes and found a high HCV seropositivity (5.4% in men, 11.5% in women) also among participants whose ALT levels were normal (79.4% in men and 90.2% in women). Furthermore, in 1999 there was no widespread awareness of the possible danger of acupuncture in the general population in Korea and the level of use of acupuncture we report, is consistent with a previous survey (Shin *et al*, 2000). A confounding effect of parenteral treatments other than acupuncture and blood transfusion is difficult to rule out. Frequent users of acupuncture are likely to suffer from diseases which might have also led to frequent intra-muscular injections or hospital admissions before universal availability of disposable syringes and needles.

In conclusion, although a fraction of HCV infections among people who underwent acupuncture may be due to other routes of parenteral transmission, we did find that a history of acupuncture was very frequent in South Korea and contributes to the high proportion of HCV seropositivity that we detected, especially among women. Safer acupuncture practice (i.e., use of disposable needles) represents, therefore, a high public health priority in South Korea and requires a close monitoring of acupuncturists nationwide.
